# Robotic versus Laparoscopic Distal Pancreatectomy: A Meta-Analysis of Short-Term Outcomes

**DOI:** 10.1371/journal.pone.0151189

**Published:** 2016-03-14

**Authors:** Jia-Yu Zhou, Chang Xin, Yi-Ping Mou, Xiao-Wu Xu, Miao-Zun Zhang, Yu-Cheng Zhou, Chao Lu, Rong-Gao Chen

**Affiliations:** 1 Department of General Surgery, School of Medicine, Zhejiang University, Hangzhou 310016, Zhejiang Province, China; 2 Department Of Hepatobiliary Surgery, Yinzhou Hospital Affiliated to Medical School of Ningbo University, Ningbo 315040, Zhejiang Province, China; 3 Department of Gastroenterology & Pancreatic Surgery, Zhejiang Provincial People Hospital, Hangzhou 310016, Zhejiang Province, China; ISMETT-UPMC Italy/ University of Catania, ITALY

## Abstract

**AIM:**

To compare the safety and efficacy of robotic-assisted distal pancreatectomy (RADP) and laparoscopic distal pancreatectomy (LDP).

**METHODS:**

A literature search of PubMed, EMBASE, and the Cochrane Library database up to June 30, 2015 was performed. The following key words were used: pancreas, distal pancreatectomy, pancreatic, laparoscopic, laparoscopy, robotic, and robotic-assisted. Fixed and random effects models were applied. Study quality was assessed using the Newcastle-Ottawa Scale.

**RESULTS:**

Seven non-randomized controlled trials involving 568 patients met the inclusion criteria. Compared with LDP, RADP was associated with longer operating time, lower estimated blood loss, a higher spleen-preservation rate, and shorter hospital stay. There was no significant difference in transfusion, conversion to open surgery, R0 resection rate, lymph nodes harvested, overall complications, severe complications, pancreatic fistula, severe pancreatic fistula, ICU stay, total cost, and 30-day mortality between the two groups.

**CONCLUSION:**

RADP is a safe and feasible alternative to LDP with regard to short-term outcomes. Further studies on the long-term outcomes of these surgical techniques are required.

**Core tip:**

To date, there is no consensus on whether laparoscopic or robotic-assisted distal pancreatectomy is more beneficial to the patient. This is the first meta-analysis to compare laparoscopic and robotic-assisted distal pancreatectomy. We found that robotic-assisted distal pancreatectomy was associated with longer operating time, lower estimated blood loss, a higher spleen-preservation rate, and shorter hospital stay. There was no significant difference in transfusion, conversion to open surgery, overall complications, severe complications, pancreatic fistula, severe pancreatic fistula, ICU stay, total cost, and 30-day mortality between the two groups.

## Introduction

Laparoscopic surgery represents one of the most important evolutions in surgical treatment in recent years. Laparoscopic distal pancreatectomy (LDP) is increasingly performed for pancreatic surgery at several specialized surgical institutions worldwide[1,2]. The conventional laparoscopic approach has many advantages such as shorter hospital stay, reduced analgesic requirement, and fewer wound infections[3]. However, this approach also has several disadvantages such as limited range of motion and the fulcrum effect which reverses movements for the surgeon in laparoscopic surgery which is eliminated in robotic surgery just as in open surgery. In order to compensate for these disadvantages, a surgical robotic system was introduced[4,5].

According to recent reports, the number of surgical procedures performed with robotic assistance has increased sharply[6,7]. However, compared with some disciplines, pancreatic surgery has been slow to adopt minimal access techniques[8]. There are some barriers to the implementation of robotic-assisted distal pancreatectomy (RADP), including the location of the pancreas and the proximity of vascular structures.

Many studies have evaluated RADP and LDP in terms of safety and efficacy, but no uniform conclusion has been reached. In the present study, we systematically reviewed the literature and conducted a meta-analysis of the reported outcomes of RADP compared with LDP to provide evidence for clinical practice.

## Materials and Methods

### Study selection

A systematic search of the literature from the Cochrane Library, PUBMED, and MEDLINE databases published between January 1992 and June 2015 was performed. The following search terms were used: pancreas, distal pancreatectomy, pancreatic, laparoscopic, laparoscopy, robotic, and robotic-assisted. A manual search was also carried out.

### Inclusion and exclusion criteria

Two reviewers (Jia-Yu Zhou and Chang Xin) retrieved eligible articles for potential studies. The inclusion criteria were: (1) papers are written in English; and (2) RADP was compared with conventional LDP. Abstracts, case reports, reviews, low-quality studies and non-comparative studies, and intraoperative data which were unable to be extracted from the published studies were excluded.

### Outcomes of interest

The following data were used to compare patients undergoing RADP with those undergoing LDP: patient characteristics, operative outcomes, and postoperative recovery. Postoperative pancreatic fistula (POPF) was defined according to the International Study Group on Pancreatic Fistula (ISGPF).

### Quality assessment

The quality of the included studies was assessed using the Newcastle-Ottawa Scale, and studies achieving six or more points were considered to be of high quality.

### Statistical analysis

This analysis was performed using Review Manager (RevMan) version 5.3. Continuous variables were evaluated by the weighted mean difference (WMD) with a 95% confidence interval (95%CI), and dichotomous variables were evaluated using odds ratios (OR) with a 95%CI. Heterogeneity was assessed using *X*^*2*^ and the *I*^*2*^ index. The fixed effect model (FEM) and random effect model (REM) were used based on the value of *I*^*2*^. *I*^*2*^ >50% was considered to show significant heterogeneity and a REM was adopted. *P*<0.05 was considered statistically significant. The statistical methods used in this study were reviewed by Ren-ChaoZhangfrom the School of Medicine, Zhejiang University.

## Results

### Description of trials and patient characteristics

**Fig 1:**
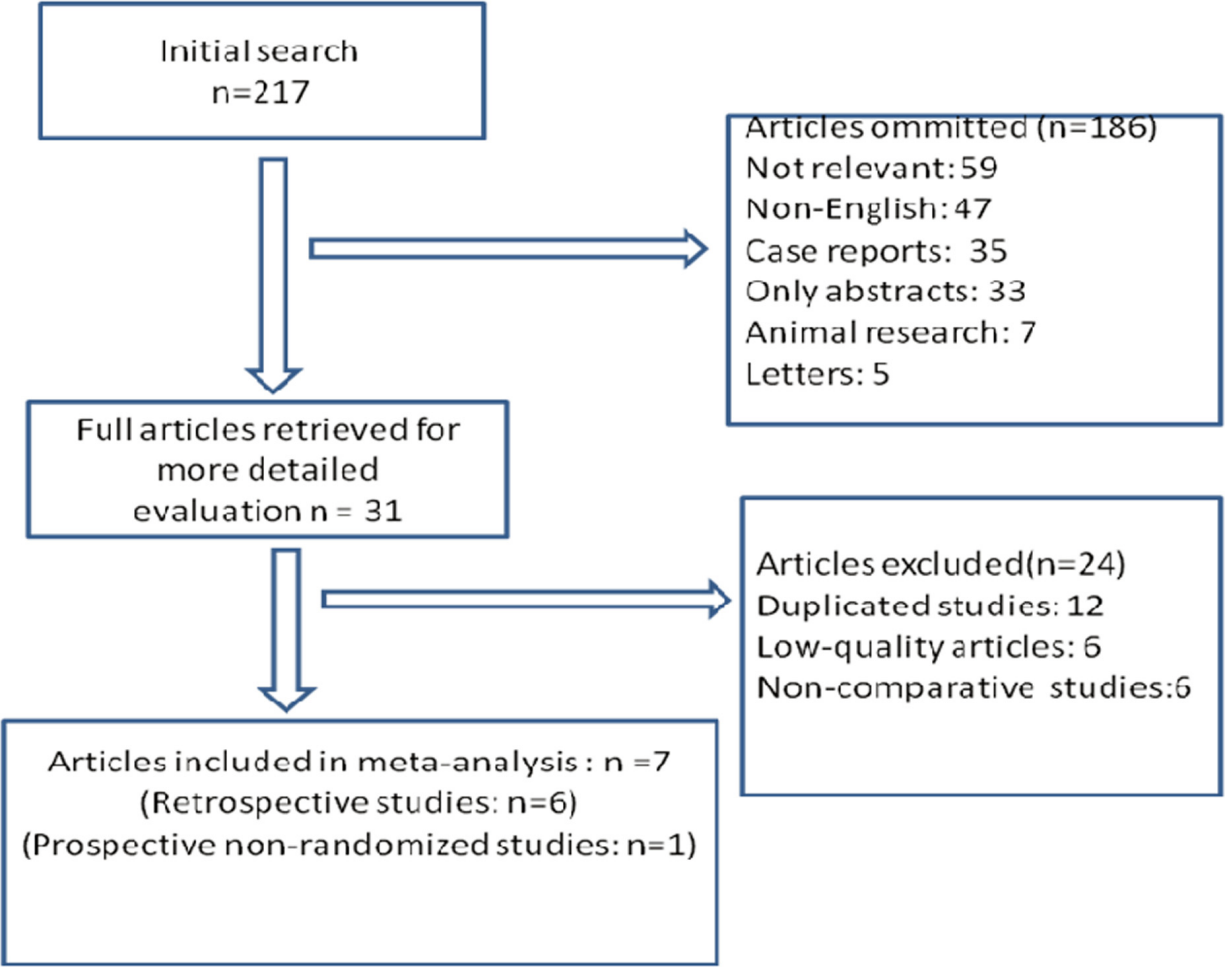
Flow chart of the selection process.

The first search strategy generated 217 studies. Only 7 articles[9–15] met the inclusion criteria. One was a prospective non-randomized study and the others were retrospective studies. The selection process is shown in Fig 1. The study characteristics and study quality are shown in Table 1. All the studies were of high quality according to the Newcastle-Ottawa Scale (NOS). A total of 568 patients were included in these studies. There were 211 patients in the RADP group and 357 patients in the LDP group. Patient characteristics in the two groups are shown in Table 2. The two groups were similar with regard to age, Body Mass Index (BMI), American Society of Anesthesiologists (ASA) score, gender, and malignant rate.

**Table 1:** Characteristics of the included studies.

Author	Year	Country	Study design	RADP *n*	LDP *n*	Study quality (score)
Waters[9]	2010	USA	Retrospective	17	18	******
Kang[10]	2011	Korea	Retrospective	20	25	******
Daouadi[11]	2013	USA	Retrospective	30	94	******
Duran[12]	2014	Spain	Retrospective	16	18	******
Lee[13]	2014	USA	Retrospective	37	131	******
Chen[14]	2015	China	Retrospective	69	50	******
Butturini[15]	2015	Italy	Prospective	22	21	*******

**Table 2:** Patient characteristics.

Study	Age(yr)	Female(%)	ASA(mean)	BMI(mean)	Malignant(%)
	RADP/ LDP	RADP VS. LDP	RADP VS. LDP	RADP VS. LDP	RADP VS. LDP
Waters[9]	64/59	*65/50 P* = 0.38	*2*.*9/2*.*8 P* = NS	NR	*0/11*.*1 P* = 0.29	
Kang[10]	44.5/56.5	*60/56 P* = NS	NR	*24*.*1/23*.*4 P* = 0.34	*P* = NS	
Daouadi[11]	59/59	*67/65 P* = 0.86	*2*.*9/3*.*2 P* = 0.8	*27*.*9/29*.*0 P* = 0.438	*P* = NS	
Duran[12]	61/58.3	*44/50 P* = NS	*2/1*.*9 P* = NS	NR	*56/44*.*4 P* = 0.49	
Lee[13]	58/58	*73/56 P* = 0.07	*2*.*5/3 P* = NS	*28*.*7/28*.*2 P* = 0.26	*10*.*8/14*.*5 P* = 0.57	
Chen[14]	56.2/56.5	*67/64 P* = 0.763	*1*.*9/1*.*94 P* = 0.989	*24*.*6/24*.*6 P* = 0.960	*23*.*2/22 P* = 0.88	
Butturini[15]	54/55	*77/71 P* = 0.929	*1*.*91/1*.*76 P* = 0.573	*25*.*33/24*.*19 P* = 0.263	*13*.*6/9*.*5 P* = 0.68	

NR: Not reported; NS: Not significant.

### Surgical outcomes

The intraoperative and postoperative outcomes are summarized in Table 3. Operative time was reported in all studies. The meta-analysis showed a statistically significant difference in operation time between the two groups (*P* = 0.02). Five studies reported estimated blood loss in the RADP and LDP groups. Analysis of the pooled data revealed that intraoperative blood loss differed significantly between the two groups with a significant level of heterogeneity (*P* = 0.01, *I*^*2*^ = 93%). Six studies presented results on spleen-preservation rate. The meta-analysis indicated that RADP had a higher spleen-preservation rate than LDP with low heterogeneity (*P*<0.00001, *I*^*2*^ = 2%).

**Table 3:** Results of the meta-analysis regarding perioperative outcome.

Perioperative outcome	No. of studies	OR/WMD	*P* value	95%CI	*I*^*2*^(%)
Surgical outcomes					
Operation time	7	45.90	0.0001	8.03,88.37	86
Blood loss	5	-185.47	0.010	-326.48,44.45	93
Blood transfusion	5	0.83	0.62	1.41,1.70	39
Spleen-preservation rate	6	3.01	0.0001	1.92,4.73	2
Conversion rate	7	0.69	0.44	0.27,1.77	50
R0 resection rate	5	6.55	0.10	0.70,60.92	0
Lymph nodes harvested	5	1.94	0.22	-1.15,5.03	91
Postoperative outcomes					
Overall complications	7	0.83	0.35	0.57,1.22	0
Severe complications	5	1.62	0.07	0.96,2.72	28
Pancreatic fistula	6	0.92	0.71	0.58,1.45	0
Severe pancreatic fistula	4	1.07	0.86	0.53,2.07	0
ICU stay	2	0.89	0.85	0.27,2.98	0
30-day mortality	7	0.51	0.35	0.12,2.12	0
Hospital stay	2	-1.14	0.01	-2.06,-0.23	49
Total cost	2	0.90	0.68	-3.38,5.51	98

OR: odds ratios; WMD: weighted mean difference

**Fig 2:**
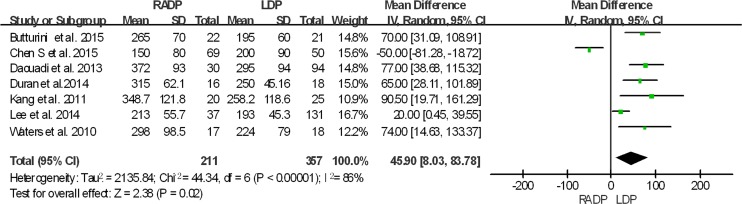
Forest plot showing the results of the meta-analysis regarding operative time.

**Fig 3:**
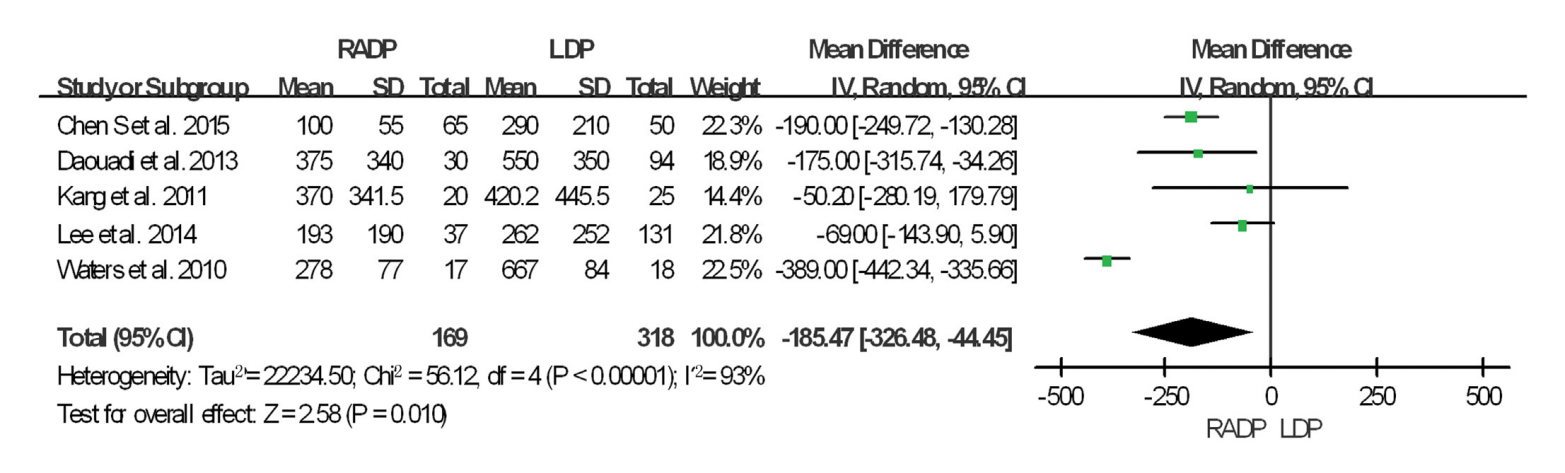
Forest plot showing the results of the meta-analysis regarding blood loss.

**Fig 4:**
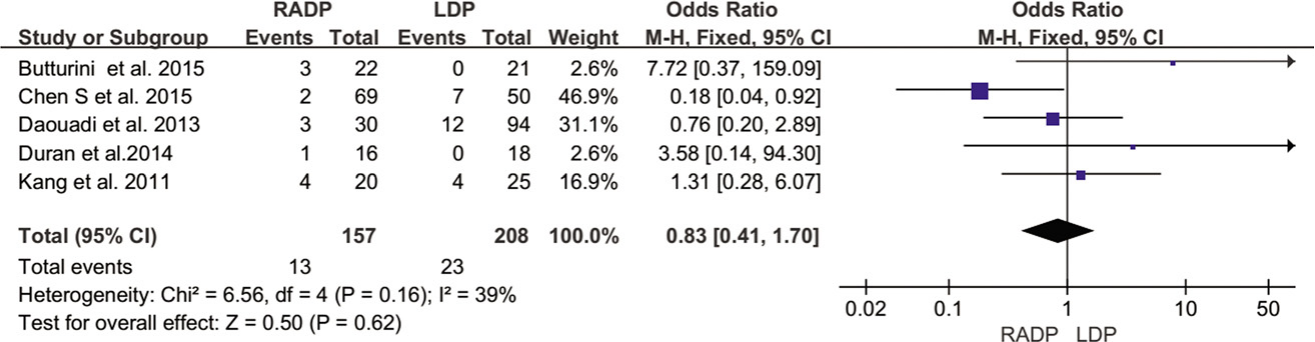
Forest plot showing the results of the meta-analysis regarding blood transfusion.

**Fig 5:**
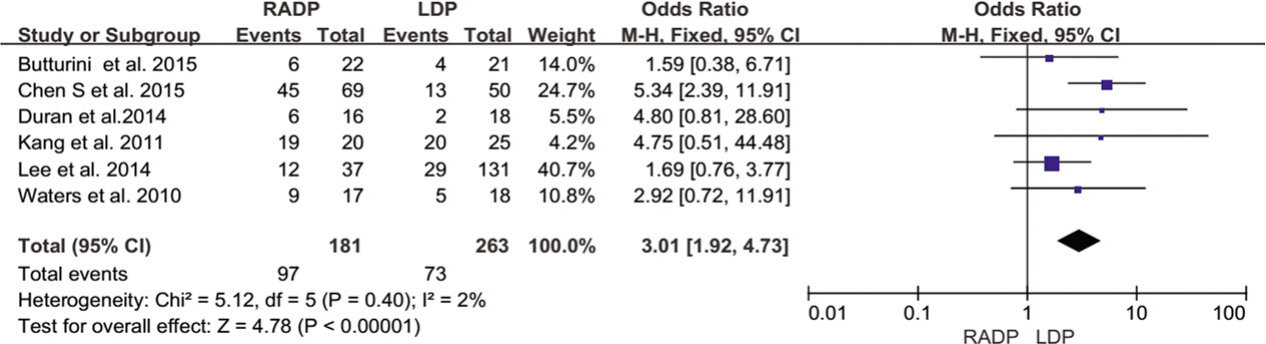
Forest plot showing the results of the meta-analysis regarding spleen-preservation rate.

**Fig 6:**
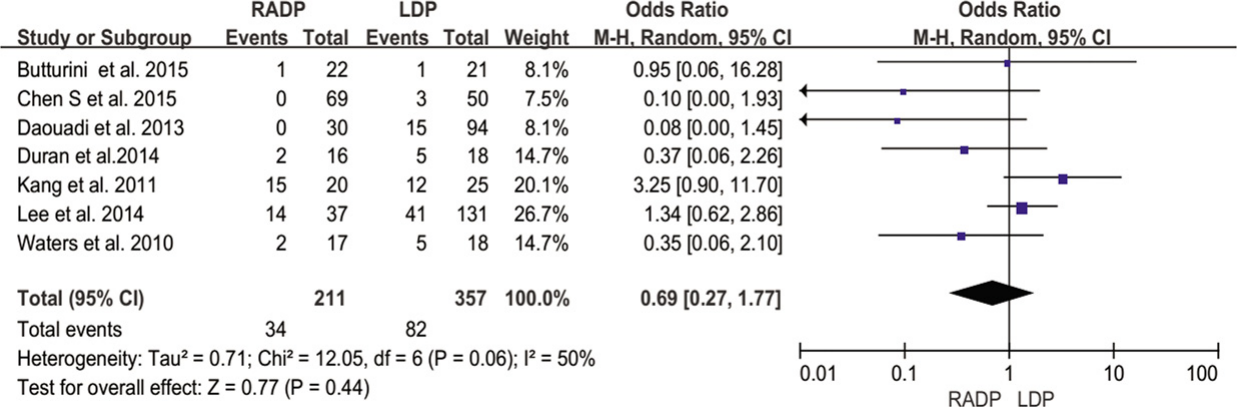
Forest plot showing the results of the meta-analysis regarding conversion rate.

**Fig 7:**
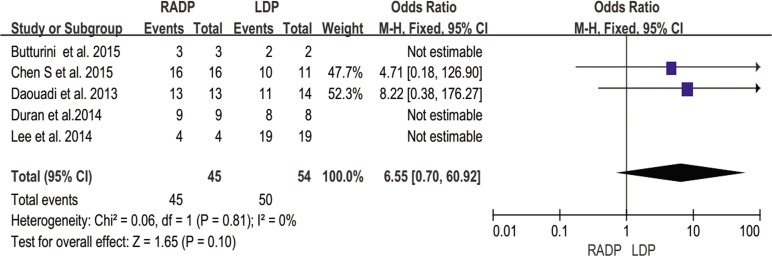
Forest plot showing the results of the meta-analysis regarding R0 resection rate.

**Fig 8:**
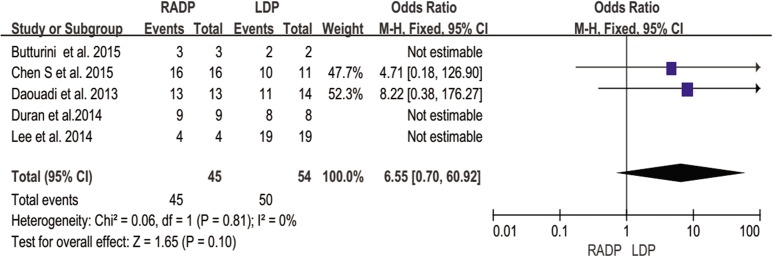
Forest plot showing the results of the meta-analysis regarding lymph nodes harvested.

In addition, no statistically significant differences in conversion to open surgery, transfusion, R0 resection rate as well as lymph nodes harvested were observed between the two groups (*P* = 0.44, *P* = 0.62, *P* = 0.10, *P* = 0.22 respectively). A Forest plot of surgical outcomes is shown in Figs 2–8.

### Postoperative recovery

#### Overall complication rate

**Fig 9:**
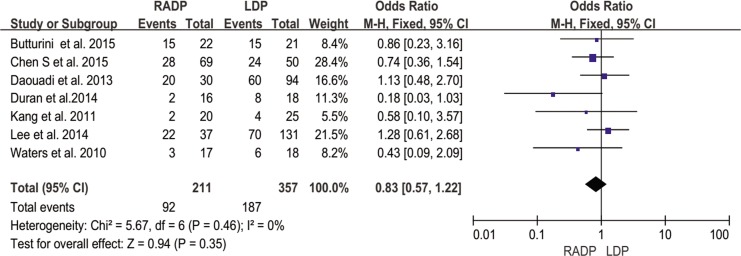
Forest plot showing the results of the meta-analysis regarding overall complications.

Five studies reported the overall complication rate. According to the results of the meta-analysis, the incidence of short-term postoperative complications was not significantly different between the two groups (*P* = 0.35), with low heterogeneity (*I*^*2*^ = 0%) in the FEM (Fig 9).

#### Severe complications (Clavien–Dindo classification > III)

**Fig 10:**
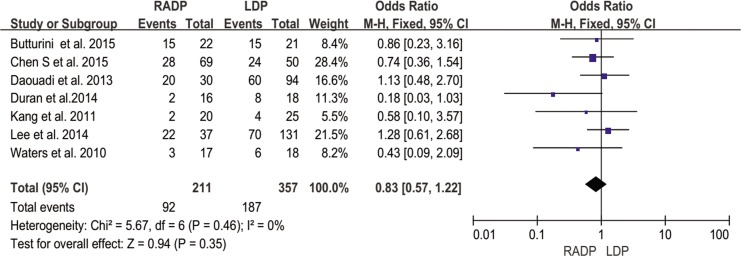
Forest plot showing the results of the meta-analysis regarding severe complications.

Severe complications were defined based on the Clavien–Dindo classification[16]. Five of the included studies recorded severe complications. The results of the meta-analysis showed no statistically significant difference between the two groups (*P* = 0.07, *I*^*2*^ = 28%) (Fig 10).

#### Pancreatic fistula

**Fig 11:**
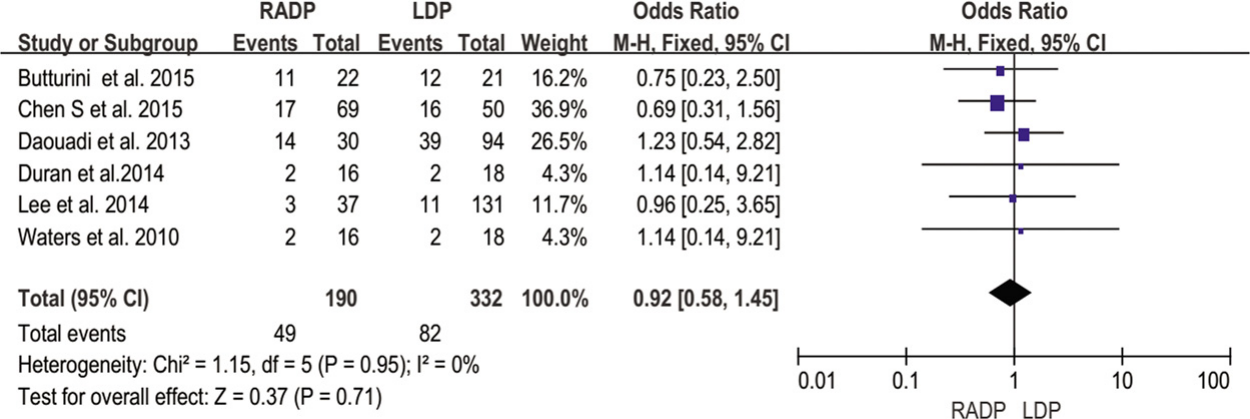
Forest plot showing the results of the meta-analysis regarding pancreatic fistula.

The study by Kang *et al* did not report data on pancreatic fistula rate. The meta-analysis showed no significant difference in the rate of pancreatic fistula with low heterogeneity (*P* = 0.71, I^2^ = 0%) (Fig 11).

#### Severe pancreatic fistula

**Fig 12:**
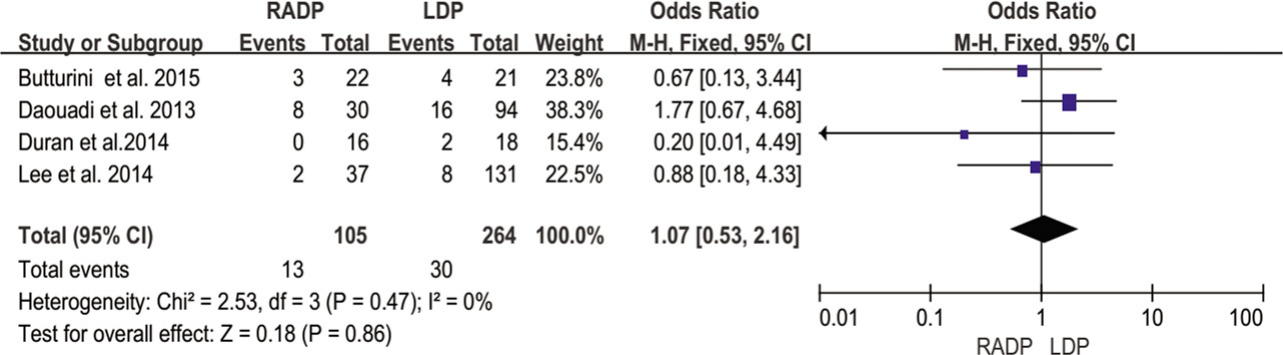
Forest plot showing the results of the meta-analysis regarding severe pancreatic fistula.

According to the ISGPF[17], severe pancreatic fistula is defined as grade B and above. Four of seven studies reported the incidence of severe pancreatic fistula and three provided incomplete data. No significant difference was found between the RADP group and the LDP group (*P* = 0.86). Heterogeneity between the two groups was low (*I*^*2*^ = 0%) (Fig 12).

#### ICU stay

**Fig 13:**
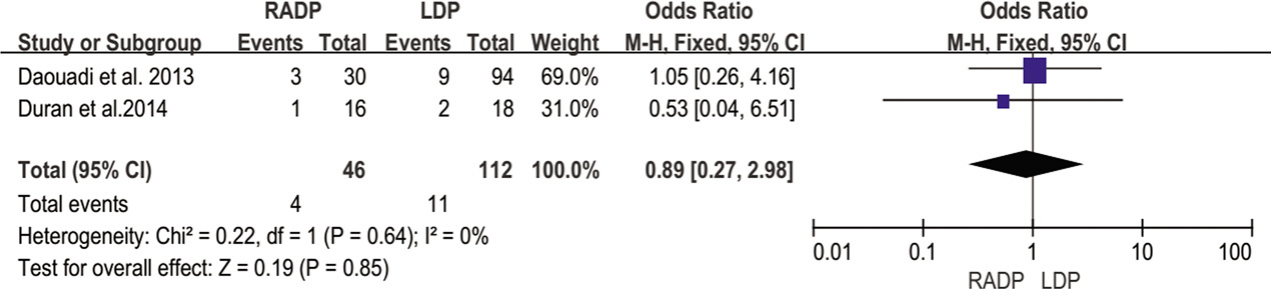
Forest plot showing the results of the meta-analysis regarding ICU stay.

Only two studies by Daouadi and Duran reporting ICU stay were included in the meta-analysis. The results showed low heterogeneity (I^2^ = 0%) between the two surgical approaches. No statistically significant difference was observed (*P* = 0.85) (Fig 13).

#### Thirty-day mortality

**Fig 14:**
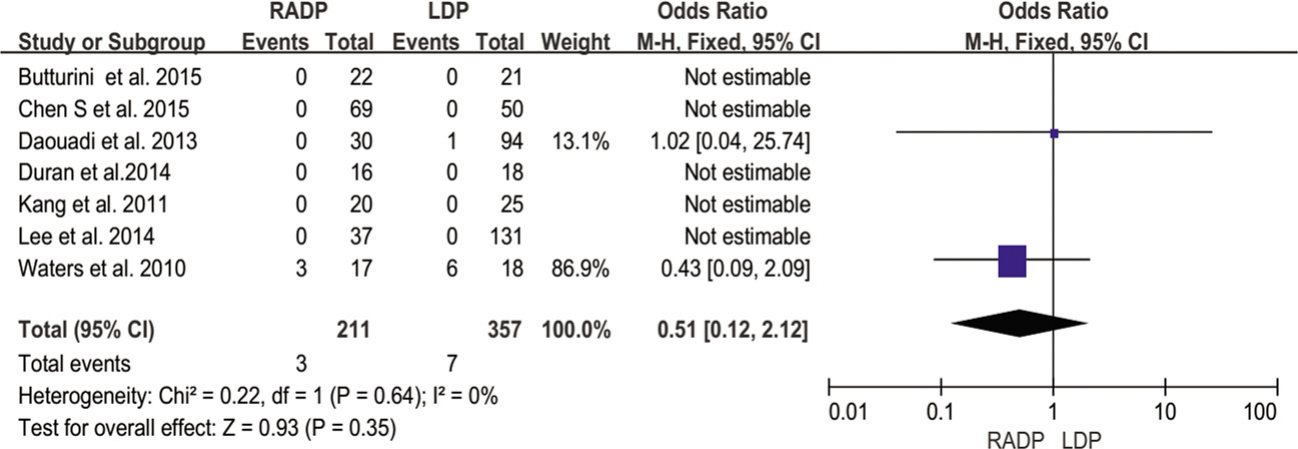
Forest plot showing the results of the meta-analysis regarding 30-day mortality.

All included studies showed a very low incidence of mortality, with no heterogeneity (*I*^*2*^ = 0%). The meta-analysis of RADP and LDP indicated a similar postoperative mortality rate (*P* = 0.35) (Fig 14).

#### Length of hospital stay

**Fig 15:**
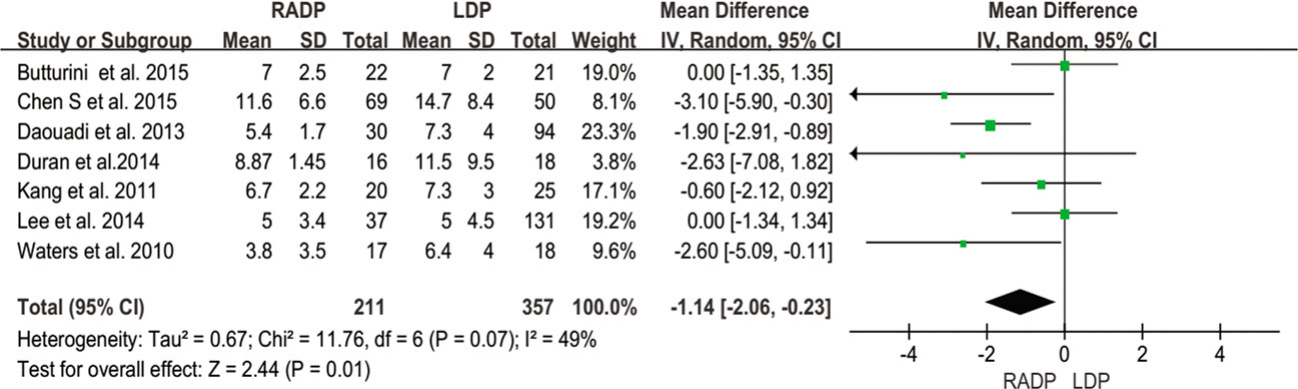
Forest plot showing the results of the meta-analysis regarding hospital stay.

Length of hospital stay was reported in all studies. A statistically significant difference was observed between the two surgical techniques. Hospital stay in the RADP group was shorter than that in the LDP group (*P* = 0.01) in the REM (Fig 15).

#### Total cost

**Fig 16:**
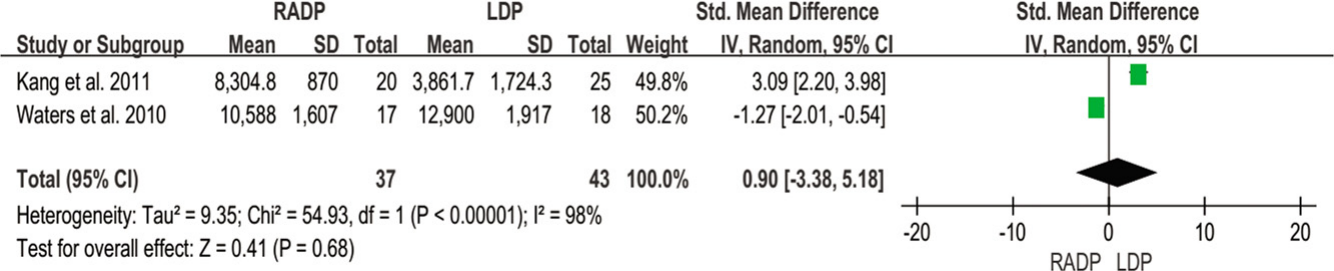
Forest plot showing the results of the meta-analysis regarding total cost.

Only two studies recorded total cost and were included in this meta-analysis. No statistically significant difference was found (*P* = 0.68) (Fig 16).

#### Publication bias

A Funnel plot analysis of total postoperative complications was performed. It was shown that none of the studies were outside the limits of 95%CI, and there was no evidence of publication bias (S1 Fig).

## Discussion

This meta-analysis of RADP and LDP demonstrated the safety and feasibility of the robotic approach. The pooled results of the seven case-control studies showed no differences in postoperative complications, 30-day mortality, ICU stay, and conversion rate between the RADP group and the LDP group.

A number of previously published studies on robotic surgery including hysterectomy[18] prostatectomy[19], and cholecystectomy[20], showed that operation time was prolonged. The analysis of surgical outcomes showed that operation time was significantly longer in the RADP group. The mean operation time in the RADP and LDP was 247.8 min and 229.9 min, respectively. This finding is in accordance with previous reviews[5,21]. With regard to the prolonged operation time with robotic surgery, there are two possible causes for this increase. Firstly, the robotic set-up often takes half an hour to complete[4,22]. Secondly, significant heterogeneity existed. The main factor in robotic-assisted surgery is the learning curve when adopting a new approach[23,24]. Surgeons at different stages of the learning curve possess different surgical skills.

Spleen preservation and blood loss are two critical factors in the success of minimally invasive distal pancreatectomy[25]. Preservation of the spleen has a beneficial effect as it boosts the patient’s immune function[26,27]. This study indicated that RADP resulted in less blood loss and a higher rate of spleen-preservation. Palep[28] suggested that the characteristics of robotic surgical systems may contribute to these outcomes. The muscle tremor filter, a three-dimensional image and incorporates motion scaling to promote the capability of performing complex tasks such as closure of the pancreas remnant and spleen preservation. This also reflects the precise surgery involved[29]. However, the studies included in this meta-analysis did not explain the exact procedure of spleen preservation. Therefore, further studies are required.

The analysis of intraoperative parameters showed that there was no significant difference in blood transfusion between the two groups. Blood transfusion may increase the risk of recurrence in patients with malignant tumors. In addition, the rate of conversion to open surgery was similar between the two groups. This may explain the similar rate of postoperative complications between the groups. In this study, we analyzed the data of R0 resection rate and lymph nodes harvested between two groups. The results were similar which confirmed the feasibility of the robotic-assisted technique in malignant tumors.

With regard to postoperative outcomes, the rate of overall complications was similar between the two groups. In the studies by Butturini and Daouadi, the most common complication was intra-abdominal infection[11,15]. With respect to severe complications, there was no statistically significant difference between the two groups. This also demonstrated the safety of robotic surgery. More importantly, analysis of the pooled data of the included studies revealed that the 30-day mortality rate and ICU stay did not differ significantly between the RADP group and the LDP group.

Pancreatic fistula is a major problem after pancreatic surgery. Studies included in this meta-analysis defined POPF according to the International Study Group on Pancreatic Fistula (ISGPF). On the basis of clinical symptoms and interventions, severe pancreatic fistula grade B and C were observed. Six of seven studies used this standard. The study by Kang[10] did not clarify the definition of POPF. The meta-analysis revealed no significant difference in the overall pancreatic fistula rate and severe pancreatic fistula rate between the RADP group and the LDP group. This demonstrated the safety and feasibility of the robotic approach.

Hospital stay is an important evaluation index in minimally invasive surgery. In this meta-analysis, shorter hospitalization was observed in the RADP group compared to the LDP group. Enhanced recovery after robotic-assisted surgery was also observed.

Many studies have reported that the cost of robotic surgery is higher than the cost of conventional laparoscopic surgery[7,30]. However, in this meta-analysis of two studies which reported cost, no statistical difference was found between the two groups. This may be explained by the shorter hospital stay in the RADP group. Total hospitalization costs were similar between the two groups.

The results of our meta-analysis should be interpreted with caution due to several limitations. Firstly, the quality of primary studies determines the quality of the results reported. None of the studies included in this meta-analysis were randomized. However, it should be taken into consideration that it is difficult to perform a prospective, randomized study on poor patient compliance. Braham[31] reported that a meta-analysis of well-designed nonrandomized comparative studies of surgical procedures is probably as accurate as randomized controlled trials. All seven studies included in this meta-analysis were found to be high-quality studies. Secondly, regarding the significant heterogeneity observed for operative time, blood loss and cost, this may have been due to the use of random-effect models in this meta-analysis. In addition, our aim was to elucidate the value of robotic surgery on short-term outcome. Due to a lack of long-term outcomes, this may affect the comprehensiveness of robotic-assisted surgery. Therefore, further long-term follow-up studies are needed to identify the potential advantages or disadvantages of RADP.

In conclusion, the short-term perioperative outcomes of RADP were found to be comparable to those of LDP. This meta-analysis found that RADP was superior to LDP for benign and malignant pancreatic diseases in terms of blood loss, spleen-preservation rate, and hospital stay, but was associated with increased operative time. However, given the aforementioned limitations of this analysis and the lack of published NRCTs, further large, multicenter, prospective randomized controlled trials are needed to demonstrate significant quantifiable differences between these surgical techniques. Long-term follow-up should be conducted in future research. Overall, it can be concluded that RADP is a safe and feasible alternative to LDP.

## Supporting Information

S1 PRISMA checklist PRISMA Checklist. (DOC)

S1 Fig Publication bias. (DOC)

S1 Table Checklist for quality assessment and scoring of nonrandomized studies. (DOC)

S2 Table Assessment of quality of studies. (DOCX)
