# Supplementary material for: Robotic versus Laparoscopic Distal Pancreatectomy: A Meta-Analysis of Short-Term Outcomes
Source: PLoS One. 2016 Mar 14;11(3):e0151189. doi: 10.1371/journal.pone.0151189 (PMC4790929; doi:10.1371/journal.pone.0151189)
Supplement: S1 Fig — (DOC) [file pone.0151189.s002.doc]

**S1 Fig Funnel plot of a comparison of overall complications**
