# Supplementary material for: Robotic versus Laparoscopic Distal Pancreatectomy: A Meta-Analysis of Short-Term Outcomes
Source: PLoS One. 2016 Mar 14;11(3):e0151189. doi: 10.1371/journal.pone.0151189 (PMC4790929; doi:10.1371/journal.pone.0151189)
Supplement: S1 Table — (DOC) [file pone.0151189.s003.doc]

S2 Table Check list for quality assessment and scoring of nonrandomized studies.

Check list

Selection

1 Is the case deﬁnition adequate?

a) yes, with independent validation preoperatively

diagnosed by upper endoscopy and staged with

endoscopic ultrasonography and abdominopelvic

computed tomography. (If yes, one star)

b) yes, e.g. record linkage or based on self reports

c) no description

2 Representativeness of the cases

a) consecutive or obviously representative series of

cases. (If yes, one star)

b) potential for selection biases or not stated

3 Selection of controls

a) community controls (If yes, one star)

b) hospital controls

c) no description

4Deﬁnition of controls

a) no history of disease of upper abdominal surgery

(If yes, one star)

b) no description of source

Comparability

Comparability of cases and controls on the basis of the design or analysis

5 study controls for clinicopathologic characteristics, such

as age, gender, BMI (If yes, one star)

6 study controls for intraoperative outcomes and postoperative

outcomes (If yes, one star)

Exposure

7 Ascertainment of exposure

a) secure record (e.g. surgical records) (If yes, one star)

b) structured interview where blind to case/control status

(If yes, one star)

c) interview not blinded to case/control status

d) written self report or medical record only

e) no description

8 Same method of ascertainment for cases and controls

a) yes (If yes, one star)

b) no

9 Non-Response rate

a) same rate for both groups (If yes, one star)

b) non respondents described

c) rate different and no designation

<http://www.ohri.ca/programs/clinical_epidemiology/oxford.asp>.
