# Supplementary material for: Robotic versus Laparoscopic Distal Pancreatectomy: A Meta-Analysis of Short-Term Outcomes
Source: PLoS One. 2016 Mar 14;11(3):e0151189. doi: 10.1371/journal.pone.0151189 (PMC4790929; doi:10.1371/journal.pone.0151189)
Supplement: S2 Table — (DOCX) [file pone.0151189.s004.docx]

S3 Table Assessment of quality of studies.

Authors Selection Comparability Exposure Score

1 2 3 4 5 6 7 8 9

| Waters^[^[^9^](#_ENREF_9)^]^ | * * * | * | * * | | ****** |
| --- | --- | --- | --- | --- | --- |
| Kang^[^[^10^](#_ENREF_10)^]^ | * | * * | * * * | ****** | |
| Daouadi^[^[^11^](#_ENREF_11)^]^ | * * | * * | * * | ****** | |
| Duran^[^[^12^](#_ENREF_12)^]^ | * * | * | * * * | ****** | |
| Lee^[^[^13^](#_ENREF_13)^]^ | * * * | * | * * | ****** | |
| Chen^[^[^14^](#_ENREF_14)^]^ | * * | * * | * * | ****** | |
| Butturini^[^[^15^](#_ENREF_15)^]^ | * * | * * | * * * | ******* | |
